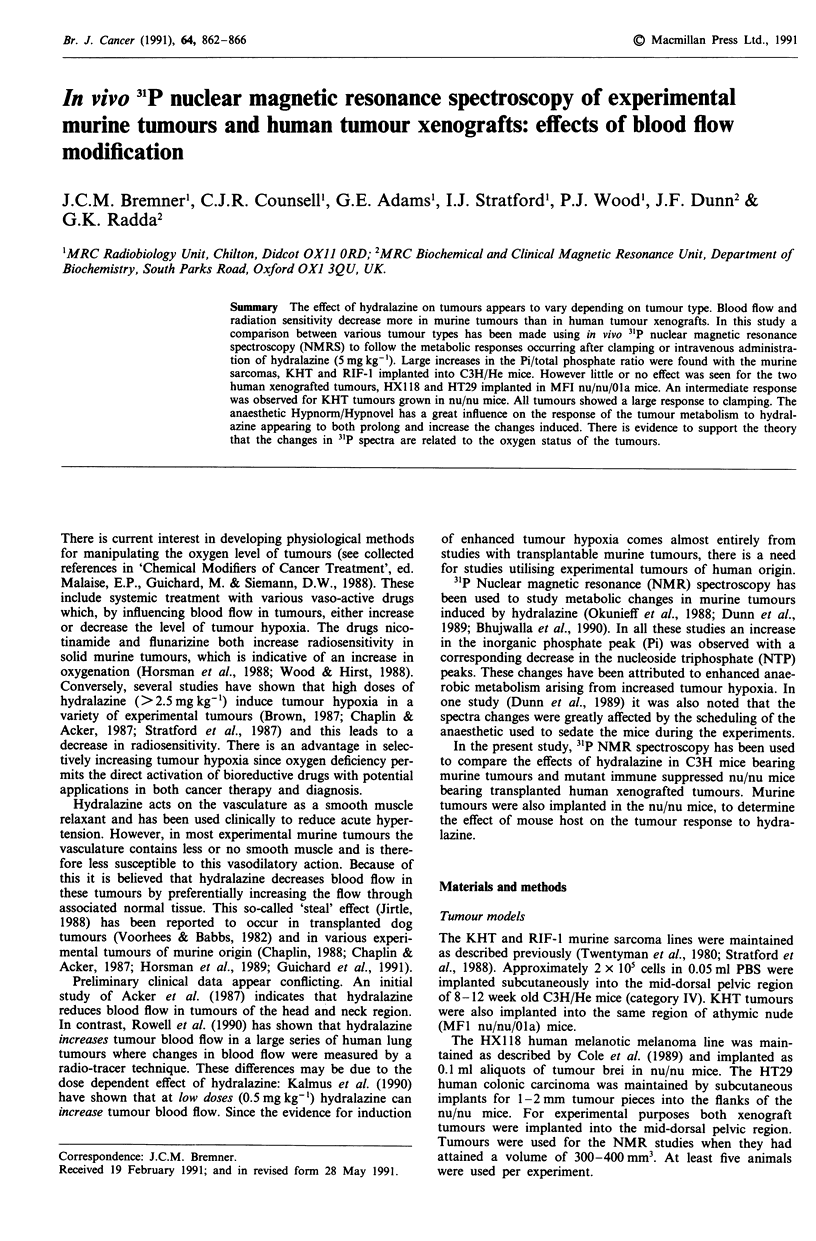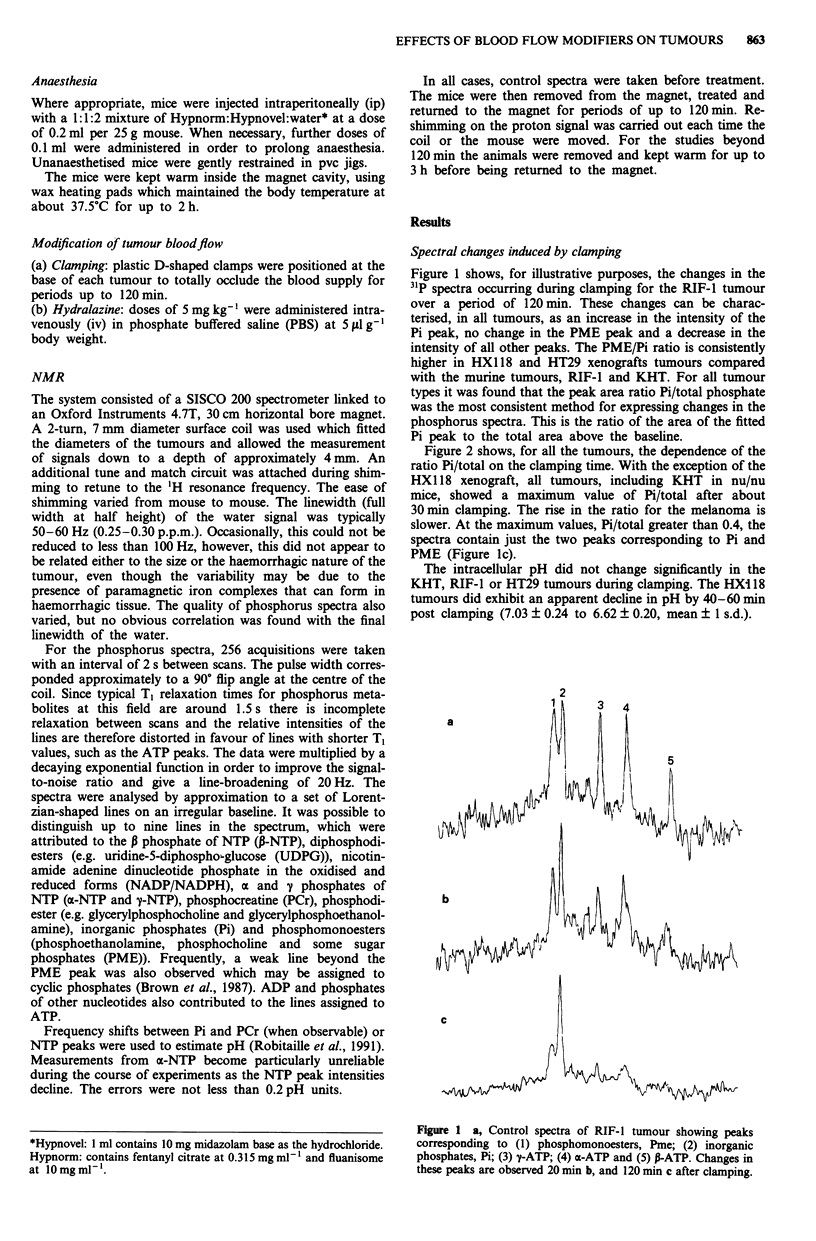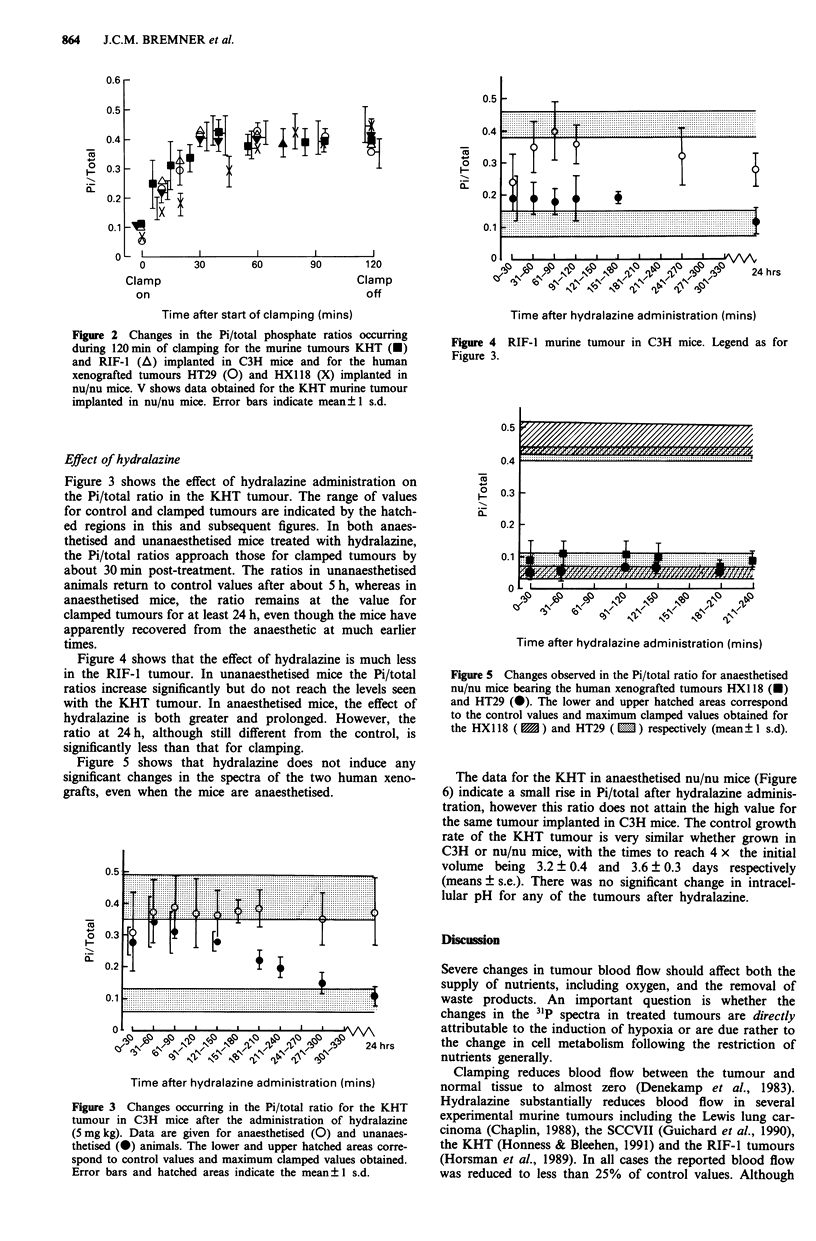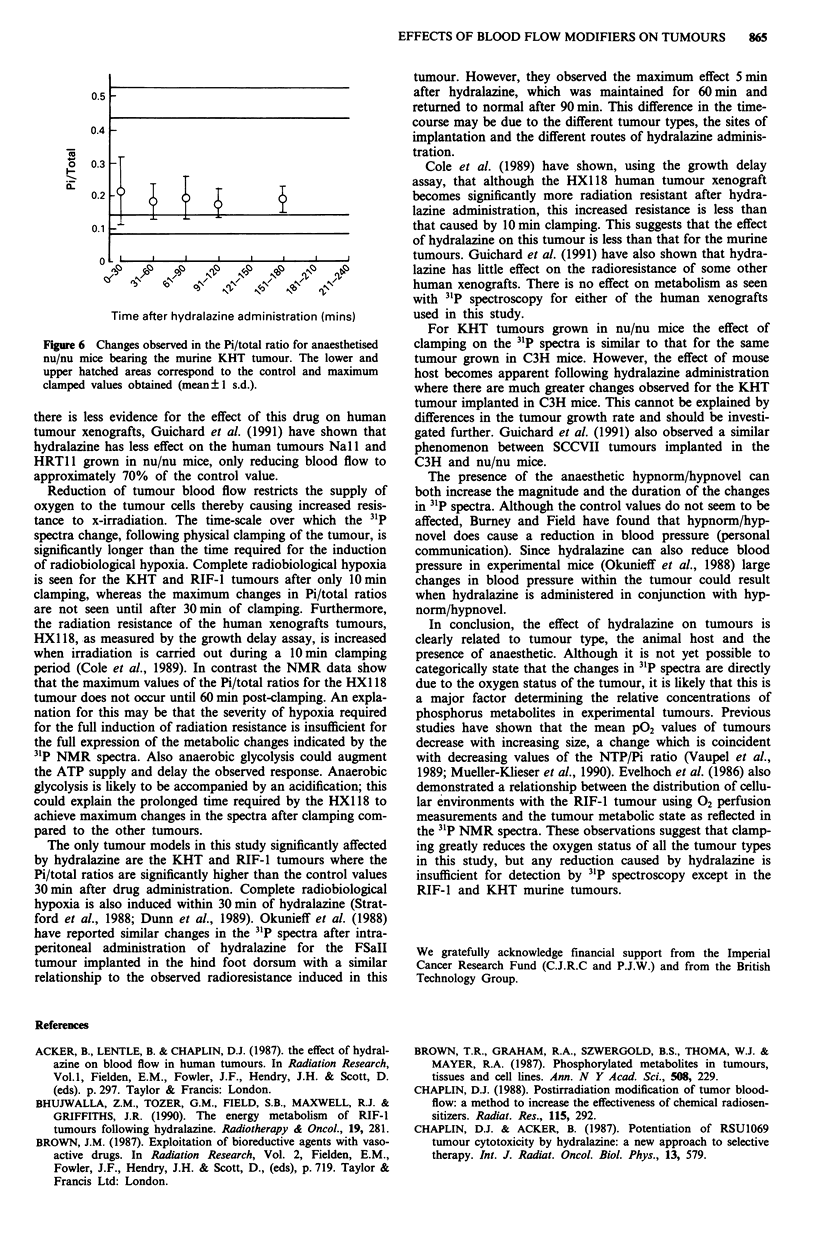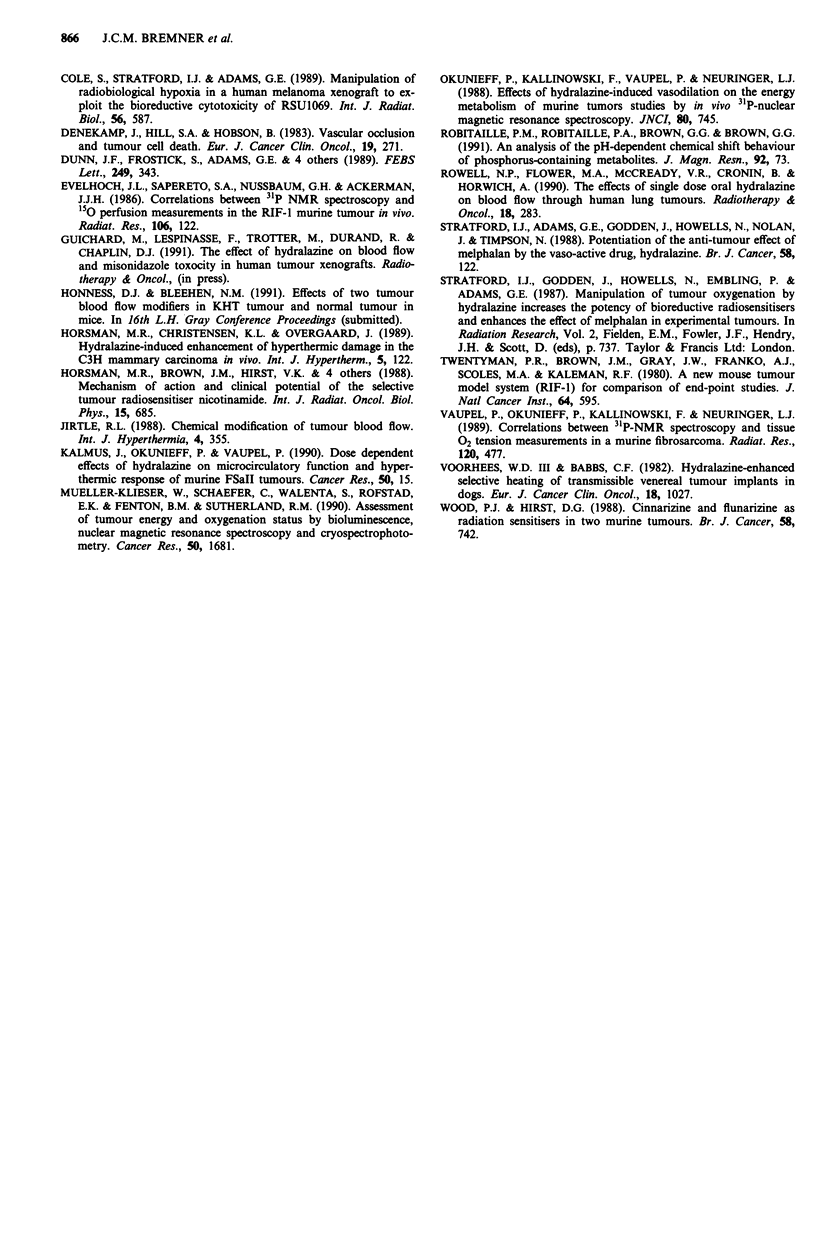# In vivo 31P nuclear magnetic resonance spectroscopy of experimental murine tumours and human tumour xenografts: effects of blood flow modification.

**DOI:** 10.1038/bjc.1991.414

**Published:** 1991-11

**Authors:** J. C. Bremner, C. J. Counsell, G. E. Adams, I. J. Stratford, P. J. Wood, J. F. Dunn, G. K. Radda

**Affiliations:** MRC Radiobiology Unit, Chilton, Didcot, UK.

## Abstract

The effect of hydralazine on tumours appears to vary depending on tumour type. Blood flow and radiation sensitivity decrease more in murine tumours than human tumour xenografts. In this study a comparison between various tumour types has been made using in vivo 31P nuclear magnetic resonance spectroscopy (NMRS) to follow the metabolic responses occurring after clamping or intravenous administration of hydralazine (5 mg kg-1). Large increases in the Pi/total phosphate ratio were found with the murine sarcomas, KHT and RIF-1 implanted into C3H/He mice. However little or no effect was seen for the two human xenografted tumours, HX118 and HT29 implanted in MFI nu/nu/01a mice. An intermediate response was observed for KHT tumours grown in nu/nu mice. All tumours showed a large response to clamping. The anaesthetic Hypnorm/Hypnovel has a great influence on the response of the tumour metabolism to hydralazine appearing to both prolong and increase the changes induced. There is evidence to support the theory that the changes in 31P spectra are related to the oxygen status of the tumours.


Br. J. Cancer (1991), 64, 862-866 © Macmillan Press Ltd., 1991

### *In vivo* [31]P nuclear magnetic resonance spectroscopy of experimental murine tumours and human tumour xenografts: effects of blood flow modification

J.C.M. Bremner[1], C.J.R. Counsell[1], G.E. Adams[1], I.J. Stratford[1], P.J. Wood[1], J.F. Dunn[2] & G.K. Radda[2]

[1]*MRC Radiobiology Unit, Chilton, Didcot OX11 0RD;* [2]*MRC Biochemical and Clinical Magnetic Resonance Unit, Department of Biochemistry, South Parks Road, Oxford OX1 3QU, UK.*

**Summary** The effect of hydralazine on tumours appears to vary depending on tumour type. Blood flow and radiation sensitivity decrease more in murine tumours than in human tumour xenografts. In this study a comparison between various tumour types has been made using *in vivo* [31]P nuclear magnetic resonance spectroscopy (NMRS) to follow the metabolic responses occurring after clamping or intravenous administration of hydralazine (5 mg $kg^{-1}$). Large increases in the Pi/total phosphate ratio were found with the murine sarcomas, KHT and RIF-1 implanted into C3H/He mice. However little or no effect was seen for the two human xenografted tumours, HX118 and HT29 implanted in MFI nu/nu/01a mice. An intermediate response was observed for KHT tumours grown in nu/nu mice. All tumours showed a large response to clamping. The anaesthetic Hypnorm/Hypnovel has a great influence on the response of the tumour metabolism to hydralazine appearing to both prolong and increase the changes induced. There is evidence to support the theory that the changes in [31]P spectra are related to the oxygen status of the tumours.

There is current interest in developing physiological methods for manipulating the oxygen level of tumours (see collected references in 'Chemical Modifiers of Cancer Treatment', ed. Malaise, E.P., Guichard, M. & Siemann, D.W., 1988). These include systemic treatment with various vaso-active drugs which, by influencing blood flow in tumours, either increase or decrease the level of tumour hypoxia. The drugs nicotinamide and flunarizine both increase radiosensitivity in solid murine tumours, which is indicative of an increase in oxygenation (Horsman *et al.*, 1988; Wood & Hirst, 1988). Conversely, several studies have shown that high doses of hydralazine ($>$2.5 mg $kg^{-1}$) induce tumour hypoxia in a variety of experimental tumours (Brown, 1987; Chaplin & Acker, 1987; Stratford *et al.*, 1987) and this leads to a decrease in radiosensitivity. There is an advantage in selectively increasing tumour hypoxia since oxygen deficiency permits the direct activation of bioreductive drugs with potential applications in both cancer therapy and diagnosis.

Hydralazine acts on the vasculature as a smooth muscle relaxant and has been used clinically to reduce acute hypertension. However, in most experimental murine tumours the vasculature contains less or no smooth muscle and is therefore less susceptible to this vasodilatory action. Because of this it is believed that hydralazine decreases blood flow in these tumours by preferentially increasing the flow through associated normal tissue. This so-called 'steal' effect (Jirtle, 1988) has been reported to occur in transplanted dog tumours (Voorhees & Babbs, 1982) and in various experimental tumours of murine origin (Chaplin, 1988; Chaplin & Acker, 1987; Horsman *et al.*, 1989; Guichard *et al.*, 1991).

Preliminary clinical data appear conflicting. An initial study of Acker *et al.* (1987) indicates that hydralazine reduces blood flow in tumours of the head and neck region. In contrast, Rowell *et al.* (1990) has shown that hydralazine *increases* tumour blood flow in a large series of human lung tumours where changes in blood flow were measured by a radio-tracer technique. These differences may be due to the dose dependent effect of hydralazine: Kalmus *et al.* (1990) have shown that at *low doses* (0.5 mg $kg^{-1}$) hydralazine can *increase* tumour blood flow. Since the evidence for induction of enhanced tumour hypoxia comes almost entirely from studies with transplantable murine tumours, there is a need for studies utilising experimental tumours of human origin.

[31]P Nuclear magnetic resonance (NMR) spectroscopy has been used to study metabolic changes in murine tumours induced by hydralazine (Okunieff *et al.*, 1988; Dunn *et al.*, 1989; Bhujwalla *et al.*, 1990). In all these studies an increase in the inorganic phosphate peak (Pi) was observed with a corresponding decrease in the nucleoside triphosphate (NTP) peaks. These changes have been attributed to enhanced anaerobic metabolism arising from increased tumour hypoxia. In one study (Dunn *et al.*, 1989) it was also noted that the spectra changes were greatly affected by the scheduling of the anaesthetic used to sedate the mice during the experiments.

In the present study, [31]P NMR spectroscopy has been used to compare the effects of hydralazine in C3H mice bearing murine tumours and mutant immune suppressed nu/nu mice bearing transplanted human xenografted tumours. Murine tumours were also implanted in the nu/nu mice, to determine the effect of mouse host on the tumour response to hydralazine.

### Materials and methods

### *Tumour models*

The KHT and RIF-1 murine sarcoma lines were maintained as described previously (Twentyman *et al.*, 1980; Stratford *et al.*, 1988). Approximately $2 \times 10^5$ cells in 0.05 ml PBS were implanted subcutaneously into the mid-dorsal pelvic region of 8–12 week old C3H/He mice (category IV). KHT tumours were also implanted into the same region of athymic nude (MF1 nu/nu/01a) mice.

The HX118 human melanotic melanoma line was maintained as described by Cole *et al.* (1989) and implanted as 0.1 ml aliquots of tumour brei in nu/nu mice. The HT29 human colonic carcinoma was maintained by subcutaneous implants for 1–2 mm tumour pieces into the flanks of the nu/nu mice. For experimental purposes both xenograft tumours were implanted into the mid-dorsal pelvic region. Tumours were used for the NMR studies when they had attained a volume of 300–400 $mm^3$. At least five animals were used per experiment.

Correspondence: J.C.M. Bremner.
Received 19 February 1991; and in revised form 28 May 1991.

*Anaesthesia*

Where appropriate, mice were injected intraperitoneally (ip) with a 1:1:2 mixture of Hypnorm:Hypnovel:water* at a dose of 0.2 ml per 25 g mouse. When necessary, further doses of 0.1 ml were administered in order to prolong anaesthesia. Unanaesthetised mice were gently restrained in pvc jigs.

The mice were kept warm inside the magnet cavity, using wax heating pads which maintained the body temperature at about 37.5°C for up to 2 h.

*Modification of tumour blood flow*

(a) *Clamping*: plastic D-shaped clamps were positioned at the base of each tumour to totally occlude the blood supply for periods up to 120 min.
(b) *Hydralazine*: doses of 5 mg $kg^{-1}$ were administered intravenously (iv) in phosphate buffered saline (PBS) at 5 $\mu l\ g^{-1}$ body weight.

*NMR*

The system consisted of a SISCO 200 spectrometer linked to an Oxford Instruments 4.7T, 30 cm horizontal bore magnet. A 2-turn, 7 mm diameter surface coil was used which fitted the diameters of the tumours and allowed the measurement of signals down to a depth of approximately 4 mm. An additional tune and match circuit was attached during shimming to retune to the $^{1}H$ resonance frequency. The ease of shimming varied from mouse to mouse. The linewidth (full width at half height) of the water signal was typically 50–60 Hz (0.25–0.30 p.p.m.). Occasionally, this could not be reduced to less than 100 Hz, however, this did not appear to be related either to the size or the haemorrhagic nature of the tumour, even though the variability may be due to the presence of paramagnetic iron complexes that can form in haemorrhagic tissue. The quality of phosphorus spectra also varied, but no obvious correlation was found with the final linewidth of the water.

For the phosphorus spectra, 256 acquisitions were taken with an interval of 2 s between scans. The pulse width corresponded approximately to a 90° flip angle at the centre of the coil. Since typical $T_1$ relaxation times for phosphorus metabolites at this field are around 1.5 s there is incomplete relaxation between scans and the relative intensities of the lines are therefore distorted in favour of lines with shorter $T_1$ values, such as the ATP peaks. The data were multiplied by a decaying exponential function in order to improve the signal-to-noise ratio and give a line-broadening of 20 Hz. The spectra were analysed by approximation to a set of Lorentzian-shaped lines on an irregular baseline. It was possible to distinguish up to nine lines in the spectrum, which were attributed to the $\beta$ phosphate of NTP ($\beta$-NTP), diphosphodiesters (e.g. uridine-5-diphospho-glucose (UDPG)), nicotinamide adenine dinucleotide phosphate in the oxidised and reduced forms (NADP/NADPH), $\alpha$ and $\gamma$ phosphates of NTP ($\alpha$-NTP and $\gamma$-NTP), phosphocreatine (PCr), phosphodiester (e.g. glycerylphosphocholine and glycerylphosphoethanolamine), inorganic phosphates (Pi) and phosphomonoesters (phosphoethanolamine, phosphocholine and some sugar phosphates (PME)). Frequently, a weak line beyond the PME peak was also observed which may be assigned to cyclic phosphates (Brown *et al.*, 1987). ADP and phosphates of other nucleotides also contributed to the lines assigned to ATP.

Frequency shifts between Pi and PCr (when observable) or NTP peaks were used to estimate pH (Robitaille *et al.*, 1991). Measurements from $\alpha$-NTP become particularly unreliable during the course of experiments as the NTP peak intensities decline. The errors were not less than 0.2 pH units.

*Hypnovel: 1 ml contains 10 mg midazolam base as the hydrochloride. Hypnorm: contains fentanyl citrate at 0.315 mg $ml^{-1}$ and fluanisome at 10 mg $ml^{-1}$.

In all cases, control spectra were taken before treatment. The mice were then removed from the magnet, treated and returned to the magnet for periods of up to 120 min. Re-shimming on the proton signal was carried out each time the coil or the mouse were moved. For the studies beyond 120 min the animals were removed and kept warm for up to 3 h before being returned to the magnet.

### Results

*Spectral changes induced by clamping*

Figure 1 shows, for illustrative purposes, the changes in the $^{31}P$ spectra occurring during clamping for the RIF-1 tumour over a period of 120 min. These changes can be characterised, in all tumours, as an increase in the intensity of the Pi peak, no change in the PME peak and a decrease in the intensity of all other peaks. The PME/Pi ratio is consistently higher in HX118 and HT29 xenografts tumours compared with the murine tumours, RIF-1 and KHT. For all tumour types it was found that the peak area ratio Pi/total phosphate was the most consistent method for expressing changes in the phosphorus spectra. This is the ratio of the area of the fitted Pi peak to the total area above the baseline.

Figure 2 shows, for all the tumours, the dependence of the ratio Pi/total on the clamping time. With the exception of the HX118 xenograft, all tumours, including KHT in nu/nu mice, showed a maximum value of Pi/total after about 30 min clamping. The rise in the ratio for the melanoma is slower. At the maximum values, Pi/total greater than 0.4, the spectra contain just the two peaks corresponding to Pi and PME (Figure 1c).

The intracellular pH did not change significantly in the KHT, RIF-1 or HT29 tumours during clamping. The HX118 tumours did exhibit an apparent decline in pH by 40–60 min post clamping (7.03 ± 0.24 to 6.62 ± 0.20, mean ± 1 s.d.).

**Figure 1** **a**, Control spectra of RIF-1 tumour showing peaks corresponding to (1) phosphomonoesters, Pme; (2) inorganic phosphates, Pi; (3) $\gamma$-ATP; (4) $\alpha$-ATP and (5) $\beta$-ATP. Changes in these peaks are observed 20 min **b**, and 120 min **c** after clamping.

**Figure 2** Changes in the Pi/total phosphate ratios occurring during 120 min of clamping for the murine tumours KHT (■) and RIF-1 (Δ) implanted in C3H mice and for the human xenografted tumours HT29 (O) and HX118 (X) implanted in nu/nu mice. V shows data obtained for the KHT murine tumour implanted in nu/nu mice. Error bars indicate mean ± 1 s.d.

*Effect of hydralazine*

Figure 3 shows the effect of hydralazine administration on the Pi/total ratio in the KHT tumour. The range of values for control and clamped tumours are indicated by the hatched regions in this and subsequent figures. In both anaesthetised and unanaesthetised mice treated with hydralazine, the Pi/total ratios approach those for clamped tumours by about 30 min post-treatment. The ratios in unanaesthetised animals return to control values after about 5 h, whereas in anaesthetised mice, the ratio remains at the value for clamped tumours for at least 24 h, even though the mice have apparently recovered from the anaesthetic at much earlier times.

Figure 4 shows that the effect of hydralazine is much less in the RIF-1 tumour. In unanaesthetised mice the Pi/total ratios increase significantly but do not reach the levels seen with the KHT tumour. In anaesthetised mice, the effect of hydralazine is both greater and prolonged. However, the ratio at 24 h, although still different from the control, is significantly less than that for clamping.

Figure 5 shows that hydralazine does not induce any significant changes in the spectra of the two human xenografts, even when the mice are anaesthetised.

**Figure 3** Changes occurring in the Pi/total ratio for the KHT tumour in C3H mice after the administration of hydralazine (5 mg kg). Data are given for anaesthetised (O) and unanaesthetised (●) animals. The lower and upper hatched areas correspond to control values and maximum clamped values obtained. Error bars and hatched areas indicate the mean ± 1 s.d.

**Figure 4** RIF-1 murine tumour in C3H mice. Legend as for Figure 3.

**Figure 5** Changes observed in the Pi/total ratio for anaesthetised nu/nu mice bearing the human xenografted tumours HX118 (■) and HT29 (●). The lower and upper hatched areas correspond to the control values and maximum clamped values obtained for the HX118 (▨) and HT29 (▩) respectively (mean ± 1 s.d).

The data for the KHT in anaesthetised nu/nu mice (Figure 6) indicate a small rise in Pi/total after hydralazine administration, however this ratio does not attain the high value for the same tumour implanted in C3H mice. The control growth rate of the KHT tumour is very similar whether grown in C3H or nu/nu mice, with the times to reach 4 × the initial volume being 3.2 ± 0.4 and 3.6 ± 0.3 days respectively (means ± s.e.). There was no significant change in intracellular pH for any of the tumours after hydralazine.

### Discussion

Severe changes in tumour blood flow should affect both the supply of nutrients, including oxygen, and the removal of waste products. An important question is whether the changes in the $^{31}P$ spectra in treated tumours are *directly* attributable to the induction of hypoxia or are due rather to the change in cell metabolism following the restriction of nutrients generally.

Clamping reduces blood flow between the tumour and normal tissue to almost zero (Denekamp *et al.*, 1983). Hydralazine substantially reduces blood flow in several experimental murine tumours including the Lewis lung carcinoma (Chaplin, 1988), the SCCVII (Guichard *et al.*, 1990), the KHT (Honness & Bleehen, 1991) and the RIF-1 tumours (Horsman *et al.*, 1989). In all cases the reported blood flow was reduced to less than 25% of control values. Although

**Figure 6** Changes observed in the Pi/total ratio for anaesthetised nu/nu mice bearing the murine KHT tumour. The lower and upper hatched areas correspond to the control and maximum clamped values obtained (mean ± 1 s.d.).

there is less evidence for the effect of this drug on human tumour xenografts, Guichard *et al.* (1991) have shown that hydralazine has less effect on the human tumours Na11 and HRT11 grown in nu/nu mice, only reducing blood flow to approximately 70% of the control value.

Reduction of tumour blood flow restricts the supply of oxygen to the tumour cells thereby causing increased resistance to x-irradiation. The time-scale over which the $^{31}P$ spectra change, following physical clamping of the tumour, is significantly longer than the time required for the induction of radiobiological hypoxia. Complete radiobiological hypoxia is seen for the KHT and RIF-1 tumours after only 10 min clamping, whereas the maximum changes in Pi/total ratios are not seen until after 30 min of clamping. Furthermore, the radiation resistance of the human xenografts tumours, HX118, as measured by the growth delay assay, is increased when irradiation is carried out during a 10 min clamping period (Cole *et al.*, 1989). In contrast the NMR data show that the maximum values of the Pi/total ratios for the HX118 tumour does not occur until 60 min post-clamping. An explanation for this may be that the severity of hypoxia required for the full induction of radiation resistance is insufficient for the full expression of the metabolic changes indicated by the $^{31}P$ NMR spectra. Also anaerobic glycolysis could augment the ATP supply and delay the observed response. Anaerobic glycolysis is likely to be accompanied by an acidification; this could explain the prolonged time required by the HX118 to achieve maximum changes in the spectra after clamping compared to the other tumours.

The only tumour models in this study significantly affected by hydralazine are the KHT and RIF-1 tumours where the Pi/total ratios are significantly higher than the control values 30 min after drug administration. Complete radiobiological hypoxia is also induced within 30 min of hydralazine (Stratford *et al.*, 1988; Dunn *et al.*, 1989). Okunieff *et al.* (1988) have reported similar changes in the $^{31}P$ spectra after intraperitoneal administration of hydralazine for the FSaII tumour implanted in the hind foot dorsum with a similar relationship to the observed radioresistance induced in this tumour. However, they observed the maximum effect 5 min after hydralazine, which was maintained for 60 min and returned to normal after 90 min. This difference in the time-course may be due to the different tumour types, the sites of implantation and the different routes of hydralazine administration.

Cole *et al.* (1989) have shown, using the growth delay assay, that although the HX118 human tumour xenograft becomes significantly more radiation resistant after hydralazine administration, this increased resistance is less than that caused by 10 min clamping. This suggests that the effect of hydralazine on this tumour is less than that for the murine tumours. Guichard *et al.* (1991) have also shown that hydralazine has little effect on the radioresistance of some other human xenografts. There is no effect on metabolism as seen with $^{31}P$ spectroscopy for either of the human xenografts used in this study.

For KHT tumours grown in nu/nu mice the effect of clamping on the $^{31}P$ spectra is similar to that for the same tumour grown in C3H mice. However, the effect of mouse host becomes apparent following hydralazine administration where there are much greater changes observed for the KHT tumour implanted in C3H mice. This cannot be explained by differences in the tumour growth rate and should be investigated further. Guichard *et al.* (1991) also observed a similar phenomenon between SCCVII tumours implanted in the C3H and nu/nu mice.

The presence of the anaesthetic hypnorm/hypnovel can both increase the magnitude and the duration of the changes in $^{31}P$ spectra. Although the control values do not seem to be affected, Burney and Field have found that hypnorm/hypnovel does cause a reduction in blood pressure (personal communication). Since hydralazine can also reduce blood pressure in experimental mice (Okunieff *et al.*, 1988) large changes in blood pressure within the tumour could result when hydralazine is administered in conjunction with hypnorm/hypnovel.

In conclusion, the effect of hydralazine on tumours is clearly related to tumour type, the animal host and the presence of anaesthetic. Although it is not yet possible to categorically state that the changes in $^{31}P$ spectra are directly due to the oxygen status of the tumour, it is likely that this is a major factor determining the relative concentrations of phosphorus metabolites in experimental tumours. Previous studies have shown that the mean $pO_2$ values of tumours decrease with increasing size, a change which is coincident with decreasing values of the NTP/Pi ratio (Vaupel *et al.*, 1989; Mueller-Klieser *et al.*, 1990). Evelhoch *et al.* (1986) also demonstrated a relationship between the distribution of cellular environments with the RIF-1 tumour using $O_2$ perfusion measurements and the tumour metabolic state as reflected in the $^{31}P$ NMR spectra. These observations suggest that clamping greatly reduces the oxygen status of all the tumour types in this study, but any reduction caused by hydralazine is insufficient for detection by $^{31}P$ spectroscopy except in the RIF-1 and KHT murine tumours.

We gratefully acknowledge financial support from the Imperial Cancer Research Fund (C.J.R.C and P.J.W.) and from the British Technology Group.

### References

ACKER, B., LENTLE, B. & CHAPLIN, D.J. (1987). the effect of hydralazine on blood flow in human tumours. In *Radiation Research*, Vol.1, Fielden, E.M., Fowler, J.F., Hendry, J.H. & Scott, D. (eds). p. 297. Taylor & Francis: London.

BHUJWALLA, Z.M., TOZER, G.M., FIELD, S.B., MAXWELL, R.J. & GRIFFITHS, J.R. (1990). The energy metabolism of RIF-1 tumours following hydralazine. *Radiotherapy & Oncol.*, **19**, 281.

BROWN, J.M. (1987). Exploitation of bioreductive agents with vasoactive drugs. In *Radiation Research*, Vol. 2, Fielden, E.M., Fowler, J.F., Hendry, J.H. & Scott, D., (eds), p. 719. Taylor & Francis Ltd: London.

BROWN, T.R., GRAHAM, R.A., SZWERGOLD, B.S., THOMA, W.J. & MAYER, R.A. (1987). Phosphorylated metabolites in tumours, tissues and cell lines. *Ann. N Y Acad. Sci.*, **508**, 229.

CHAPLIN, D.J. (1988). Postirradiation modification of tumor bloodflow: a method to increase the effectiveness of chemical radiosensitizers. *Radiat. Res.*, **115**, 292.

CHAPLIN, D.J. & ACKER, B. (1987). Potentiation of RSU1069 tumour cytotoxicity by hydralazine: a new approach to selective therapy. *Int. J. Radiat. Oncol. Biol. Phys.*, **13**, 579.

COLE, S., STRATFORD, I.J. & ADAMS, G.E. (1989). Manipulation of radiobiological hypoxia in a human melanoma xenograft to exploit the bioreductive cytotoxicity of RSU1069. *Int. J. Radiat. Biol.*, **56**, 587.

DENEKAMP, J., HILL, S.A. & HOBSON, B. (1983). Vascular occlusion and tumour cell death. *Eur. J. Cancer Clin. Oncol.*, **19**, 271.

DUNN, J.F., FROSTICK, S., ADAMS, G.E. & 4 others (1989). *FEBS Lett.*, **249**, 343.

EVELHOCH, J.L., SAPERETO, S.A., NUSSBAUM, G.H. & ACKERMAN, J.J.H. (1986). Correlations between $^{31}$P NMR spectroscopy and $^{15}$O perfusion measurements in the RIF-1 murine tumour *in vivo*. *Radiat. Res.*, **106**, 122.

GUICHARD, M., LESPINASSE, F., TROTTER, M., DURAND, R. & CHAPLIN, D.J. (1991). The effect of hydralazine on blood flow and misonidazole toxocity in human tumour xenografts. *Radiotherapy & Oncol.*, (in press).

HONNESS, D.J. & BLEEHEN, N.M. (1991). Effects of two tumour blood flow modifiers in KHT tumour and normal tumour in mice. In *16th L.H. Gray Conference Proceedings* (submitted).

HORSMAN, M.R., CHRISTENSEN, K.L. & OVERGAARD, J. (1989). Hydralazine-induced enhancement of hyperthermic damage in the C3H mammary carcinoma *in vivo*. *Int. J. Hypertherm.*, **5**, 122.

HORSMAN, M.R., BROWN, J.M., HIRST, V.K. & 4 others (1988). Mechanism of action and clinical potential of the selective tumour radiosensitiser nicotinamide. *Int. J. Radiat. Oncol. Biol. Phys.*, **15**, 685.

JIRTLE, R.L. (1988). Chemical modification of tumour blood flow. *Int. J. Hyperthermia*, **4**, 355.

KALMUS, J., OKUNIEFF, P. & VAUPEL, P. (1990). Dose dependent effects of hydralazine on microcirculatory function and hyperthermic response of murine FSaII tumours. *Cancer Res.*, **50**, 15.

MUELLER-KLIESER, W., SCHAEFER, C., WALENTA, S., ROFSTAD, E.K. & FENTON, B.M. & SUTHERLAND, R.M. (1990). Assessment of tumour energy and oxygenation status by bioluminescence, nuclear magnetic resonance spectroscopy and cryospectrophotometry. *Cancer Res.*, **50**, 1681.

OKUNIEFF, P., KALLINOWSKI, F., VAUPEL, P. & NEURINGER, L.J. (1988). Effects of hydralazine-induced vasodilation on the energy metabolism of murine tumors studies by *in vivo* $^{31}$P-nuclear magnetic resonance spectroscopy. *JNCI*, **80**, 745.

ROBITAILLE, P.M., ROBITAILLE, P.A., BROWN, G.G. & BROWN, G.G. (1991). An analysis of the pH-dependent chemical shift behaviour of phosphorus-containing metabolites. *J. Magn. Resn.*, **92**, 73.

ROWELL, N.P., FLOWER, M.A., MCCREADY, V.R., CRONIN, B. & HORWICH, A. (1990). The effects of single dose oral hydralazine on blood flow through human lung tumours. *Radiotherapy & Oncol.*, **18**, 283.

STRATFORD, I.J., ADAMS, G.E., GODDEN, J., HOWELLS, N., NOLAN, J. & TIMPSON, N. (1988). Potentiation of the anti-tumour effect of melphalan by the vaso-active drug, hydralazine. *Br. J. Cancer*, **58**, 122.

STRATFORD, I.J., GODDEN, J., HOWELLS, N., EMBLING, P. & ADAMS, G.E. (1987). Manipulation of tumour oxygenation by hydralazine increases the potency of bioreductive radiosensitisers and enhances the effect of melphalan in experimental tumours. In *Radiation Research*, Vol. 2, Fielden, E.M., Fowler, J.F., Hendry, J.H. & Scott, D. (eds), p. 737. Taylor & Francis Ltd: London.

TWENTYMAN, P.R., BROWN, J.M., GRAY, J.W., FRANKO, A.J., SCOLES, M.A. & KALEMAN, R.F. (1980). A new mouse tumour model system (RIF-1) for comparison of end-point studies. *J. Natl Cancer Inst.*, **64**, 595.

VAUPEL, P., OKUNIEFF, P., KALLINOWSKI, F. & NEURINGER, L.J. (1989). Correlations between $^{31}$P-NMR spectroscopy and tissue $O_2$ tension measurements in a murine fibrosarcoma. *Radiat. Res.*, **120**, 477.

VOORHEES, W.D. III & BABBS, C.F. (1982). Hydralazine-enhanced selective heating of transmissible venereal tumour implants in dogs. *Eur. J. Cancer Clin. Oncol.*, **18**, 1027.

WOOD, P.J. & HIRST, D.G. (1988). Cinnarizine and flunarizine as radiation sensitisers in two murine tumours. *Br. J. Cancer*, **58**, 742.